# miR-34 miRNAs Regulate Cellular Senescence in Type II Alveolar Epithelial Cells of Patients with Idiopathic Pulmonary Fibrosis

**DOI:** 10.1371/journal.pone.0158367

**Published:** 2016-06-30

**Authors:** Supparerk Disayabutr, Eun Kyung Kim, Seung-Ick Cha, Gary Green, Ram P. Naikawadi, Kirk D. Jones, Jeffrey A. Golden, Aaron Schroeder, Michael A. Matthay, Jasleen Kukreja, David J. Erle, Harold R. Collard, Paul J. Wolters

**Affiliations:** 1 Division of Pulmonary, Critical Care, Allergy and Sleep Medicine, Department of Medicine, University of California San Francisco, San Francisco, California, United States of America; 2 Department of Internal Medicine, CHA Bundang Medical Center, College of Medicine, CHA University, Seongnam, Korea; 3 Department of Internal Medicine, Kyungpook National University Hospital, Daegu, Korea; 4 Department of Pathology, University of California San Francisco, San Francisco, California, United States of America; 5 Department of Surgery, University of California San Francisco, San Francisco, California, United States of America; University of Pittsburgh, UNITED STATES

## Abstract

Pathologic features of idiopathic pulmonary fibrosis (IPF) include genetic predisposition, activation of the unfolded protein response, telomere attrition, and cellular senescence. The mechanisms leading to alveolar epithelial cell (AEC) senescence are poorly understood. MicroRNAs (miRNAs) have been reported as regulators of cellular senescence. Senescence markers including p16, p21, p53, and senescence-associated β-galactosidase (SA-βgal) activity were measured in type II AECs from IPF lungs and unused donor lungs. miRNAs were quantified in type II AECs using gene expression arrays and quantitative RT-PCR. Molecular markers of senescence (p16, p21, and p53) were elevated in IPF type II AECs. SA-βgal activity was detected in a greater percentage in type II AECs isolated from IPF patients (23.1%) compared to patients with other interstitial lung diseases (1.2%) or normal controls (0.8%). The relative levels of senescence-associated miRNAs miR-34a, miR-34b, and miR-34c, but not miR-20a, miR-29c, or miR-let-7f were significantly higher in type II AECs from IPF patients. Overexpression of miR-34a, miR-34b, or miR-34c in lung epithelial cells was associated with higher SA-βgal activity (27.8%, 35.1%, and 38.2%, respectively) relative to control treated cells (8.8%). Targets of miR-34 miRNAs, including E2F1, c-Myc, and cyclin E2, were lower in IPF type II AECs. These results show that markers of senescence are uniquely elevated in IPF type II AECs and suggest that the miR-34 family of miRNAs regulate senescence in IPF type II AECs.

## Introduction

The prevalence of idiopathic pulmonary fibrosis (IPF) is estimated to be 14 to 43 per 100,000 people in the United States [1] and increases with age ranging from 4 per 100,000 people aged 18 to 34 years to 227 per 100,000 people among those aged 75 years or older. Additionally, recent reports have demonstrated that the prevalence is increasing with aging of the population in the United States. [2] Although IPF is now recognized to be a disease associated with chronological aging, age-associated molecular changes contributing to the development or progression of IPF are incompletely understood. [3] One contributing factor may be telomere shortening, which has been found in lung epithelial cells of most IPF patients. [4, 5] Shortened peripheral blood telomeres have also been shown to predict worse outcome of IPF patients. [6]

Cellular senescence is an irreversible cell-cycle arrest that has been associated with age-related diseases including IPF. [7] Cellular senescence can be mediated by multiple stimuli including telomere shortening, DNA damage, oncogene expression, and oxidative stress. [8] Molecular changes that regulate cellular senescence include the p53-p21-pRb or the p16-pRb pathways. [9, 10] Senescent cells can be identified by the expression of these markers or senescence-associated β-galactosidase (SA-βgal) activity. [9, 11, 12]

MicroRNAs (miRNAs) are non-coding RNAs that regulate gene expression at the post-transcriptional level. miRNAs induce changes in various biological processes, including apoptosis, proliferation, and cellular senescence, by regulating expression of a variety of target genes. [13] Reports of differential expression of miRNAs in the lungs of IPF patients [14] suggest they may be involved in the pathogenesis of IPF. A number of senescence-associated miRNAs (SA-miRNAs) have been reported [15, 16] including the miR-34 family of miRNAs that are downstream effectors of p53-mediated cellular senescence. [17, 18]

In this study, we measured senescence markers, p16, p21, p53, and SA-βgal activity, in lung tissues and purified type II alveolar epithelial cells (AECs) from IPF patients and control subjects. Then we measured expression of SA-miRNAs and their target mRNAs in type II AECs from IPF lungs compared to those from normal lungs using microRNA microarrays. Finally, we confirmed SA-miRNAs regulate cellular senescence by measuring senescence markers in lung epithelial cells overexpressing differentially expressed miRNAs.

## Materials and Methods

### Subjects

IPF was established through a multidisciplinary review of clinical data, radiology, and pathology according to established criteria. [19] The diagnosis of scleroderma was based on the criteria of the American College of Rheumatology [20] and chronic hypersensitivity pneumonitis on the diagnostic criteria suggested by Hanak. [21] Diseased lung tissues were obtained at the time of lung transplantation. Non-diseased normal lung tissues were procured from lungs not used by the Northern California Transplant Donor Network. The UCSF committee on Human Research approved the study protocols and all study subjects provided written informed consent for their participation.

### Type II alveolar epithelial cell isolation

Type II AECs were isolated from explanted IPF, non-IPF interstitial lung disease, and unused donor lungs as previously described. [22] Briefly, Human alveolar epithelial type II cells were isolated from explanted lungs or human lungs not used by the Northern California Transplant Donor Network. The pulmonary artery was perfused with PBS and distal airspaces lavaged several times with PBS. Then HBSS containing elastase was instilled into distal air spaces and the lung incubated at 37°C for 60 min. The elastase-digested lung was minced in the presence of bovine serum and DNase and the cell rich fraction sequentially filtered through nylon meshes. Filtered cells were separated using a discontinuous percoll (Sigma-Aldrich, St. Louis, MO) density gradient centrifuged at 400g for 20 min. The band containing type II cells was collected, washed, then resuspended in PBS containing FCS and incubated on human IgG (Equitech, Kerrville, TX) -coated tissue-culture treated Petri dishes for up to 90 minutes. Unattached type II cells were collected and stained for Epcam (eBioscience, San Diego, CA), CD45 (Invitrogen, Life Technologies, Carlsbad, CA), and T1a (Angiobio, Del Mar, CA). The cells were then analyzed using a BD FACSAria Fusion cell sorter and the Epcam^+^, CD45^-^, T1a^-^ fraction isolated. Purity of human alveolar type II cells, assessed by pro-SPC staining on cytospun cells was ≥ 90% (S1 Fig).

### Immunohistochemistry

5-μm sections of paraffin embedded, formalin fixed, lung were stained for p16, p21, and p53 by immunohistochemistry as previously described. [23] Briefly, the sections were incubated in PBS containing 5% normal goat serum and 1% bovine serum albumin (BSA), then incubated with a 1:100 dilution of mouse anti-human p16 (Santa Cruz Biotechnology, Santa Cruz, CA), rabbit anti-human p21 (Santa Cruz Biotechnology) or mouse anti-human p53 antibody (Santa Cruz Biotechnology) overnight at 4°C. The sections were washed, incubated with horseradish peroxidase (HRP)-conjugated goat anti-mouse or anti-rabbit IgG (Santa Cruz Biotechnology). After washing, the bound peroxidase activity was detected using a diaminobenzidine (DAB) substrate kit (Vector Laboratories, Burlingame, CA).

### Immunoblots

Type II AECs were lysed in RIPA buffer and the cell lysates subjected to SDS-PAGE under reducing conditions and transferred to nitrocellulose membrane (Life Sciences Products, Boston, MA). [22] The membranes were washed with 50 mM Tris-HCl containing 0.5 M NaCl, 0.01% Tween 20, (TBS; pH 7.5) and incubated overnight in 5% milk containing primary antibody: mouse anti-human p16 (Santa Cruz Biotechnology), rabbit anti-human p21 (Santa Cruz Biotechnology), mouse anti-human p53 (Santa Cruz Biotechnology), or mouse anti-human β-actin (Santa Cruz Biotechnology). The membrane was washed with TBS, then incubated with secondary antibody and washed again. Immunoreactivity was detected using the phototope-HRP-detection kit (New England Biolabs, Beverly, MA).

### Senescence-associated β-galactosidase (SA-βgal) activity detection

SA-βgal activity was detected by using histochemical assay and fluorescence assay adapted for flow cytometry. [24] For the histochemical assay, cryosections of frozen lung tissue were fixed with 1% formaldehyde and washed in PBS then incubated overnight at 37°C with 5-bromo-4-chloro-3-indolyl β-D-galactopyranoside (X-gal, Bioline, London, UK) in a buffer at pH 6.0. After washing, the sections were counterstained with eosin and imaged using bright field microscopy. For the fluorescence assay, cells were resuspended in DMEM containing 10% fetal bovine serum (FBS) then incubated with 5-dodecanoylaminofluorescein di-β-D-galactopyranoside (C_12_FDG, Invitrogen, Life Technologies, Carlsbad, CA) for 1 hr at room temperature after lysosomal alkalinization with bafilomycin A1 (Santa Cruz Biotechnology). We further stained the cells with the following antibodies or substrates: Sytox blue dead cell stain (Invitrogen), Pacific blue-conjugated anti-human CD45 (Invitrogen), eFluor 660-conjugated anti-human CD326 (Epcam, eBioscience), anti-human podoplanin (Angiobio), and Brilliant violet 421 anti-rat IgG (Biolegend, San Diego, CA). Hematopoietic and dead cell populations were excluded by using anti-human CD45 antibody and Sytox. Then type II AEC population was selected for detection of SA-βgal activity. The cells were examined by flow cytometry with the BD LSRII flow cytometer (BD Biosciences, San Jose, CA). Data were analyzed by FACSDiva Software (BD Biosciences).

### Lentivirus infection

A549 cells were plated in DMEM with 10% FBS and allowed to adhere overnight. Lentivirus expressing miR-34a, miR-34b, or miR-34c co-expressing mCherry as transduction control in a miR-30 context [25] were added in diethylaminoethyl-Dextran and the cells were incubated for 24 hours. After 24 hours, equal amount of fresh media was added and the cells were incubated for additional 72 hours prior to staining for SA-βgal activity. The transfection efficiency was 80–95%.

### Quantitative real-time polymerase chain reaction (qRT-PCR)

Total RNA containing miRNAs were isolated from type II AECs using miRNeasy Mini Kit (Qiagen, Valencia, CA) according to manufacturer’s instruction. The miRNA and gene expression levels were quantified by qRT-PCR using Taqman Universal Master Mix Reagents (Applied Biosystems, Foster City, CA) and SensiFAST SYBR Lo-ROX kit (Bioline), respectively. The qRT-PCR was carried out on Life Technologies ViiA7 Real Time PCR system (Applied Biosystems). The miRNA expression levels were determined by the threshold cycle (C_T_) after normalization with miR-103 and miR-191 as endogenous controls. [26] The gene expression levels were determined by the C_T_ value after normalization with β-actin and GAPDH as endogenous controls. The expression levels of miRNA or gene were presented as the normalized relative log_2_ expression. The specific primers used are listed in S1 Table.

### miRNA arrays

Total RNA quality was assessed using a Pico Chip on an Agilent 2100 Bioanalyzer (Agilent Technologies, Palo Alto, CA). RNA was labeled with Cy3-CTP using the miRCURY LNA microRNA power labeling kit (Exiqon, Inc, Woburn, MA), according to manufacturers protocol. Labeled RNA was hybridized to Agilent custom UCSF miRNA v3.5 multi-species 8x15K Ink-jet arrays. Hybridizations were performed for 16 hrs, according to the manufacturers protocol. Arrays were scanned using the Agilent microarray scanner and raw signal intensities were extracted with Feature Extraction v10.1 software. This dataset was normalized using the quantile normalization method. [27] No background subtraction was performed, and the median feature pixel intensity was used as the raw signal before normalization. A one-way ANOVA linear model was fit to the comparison to estimate the mean M values and calculated moderated t-statistic, B statistic, false discovery rate and p-value for each miRNA for the comparison of interest. Since the microarray study was a screen involving a small number of subjects, we used a 2-fold difference and a nominal (unadjusted) *p* value < 0.05 to identify candidate miRNAs for further study. All procedures were carried out using functions in the R package limma in Bioconductor. [28]

## Results

### Tissue SA-βgal staining

**Fig 1:**
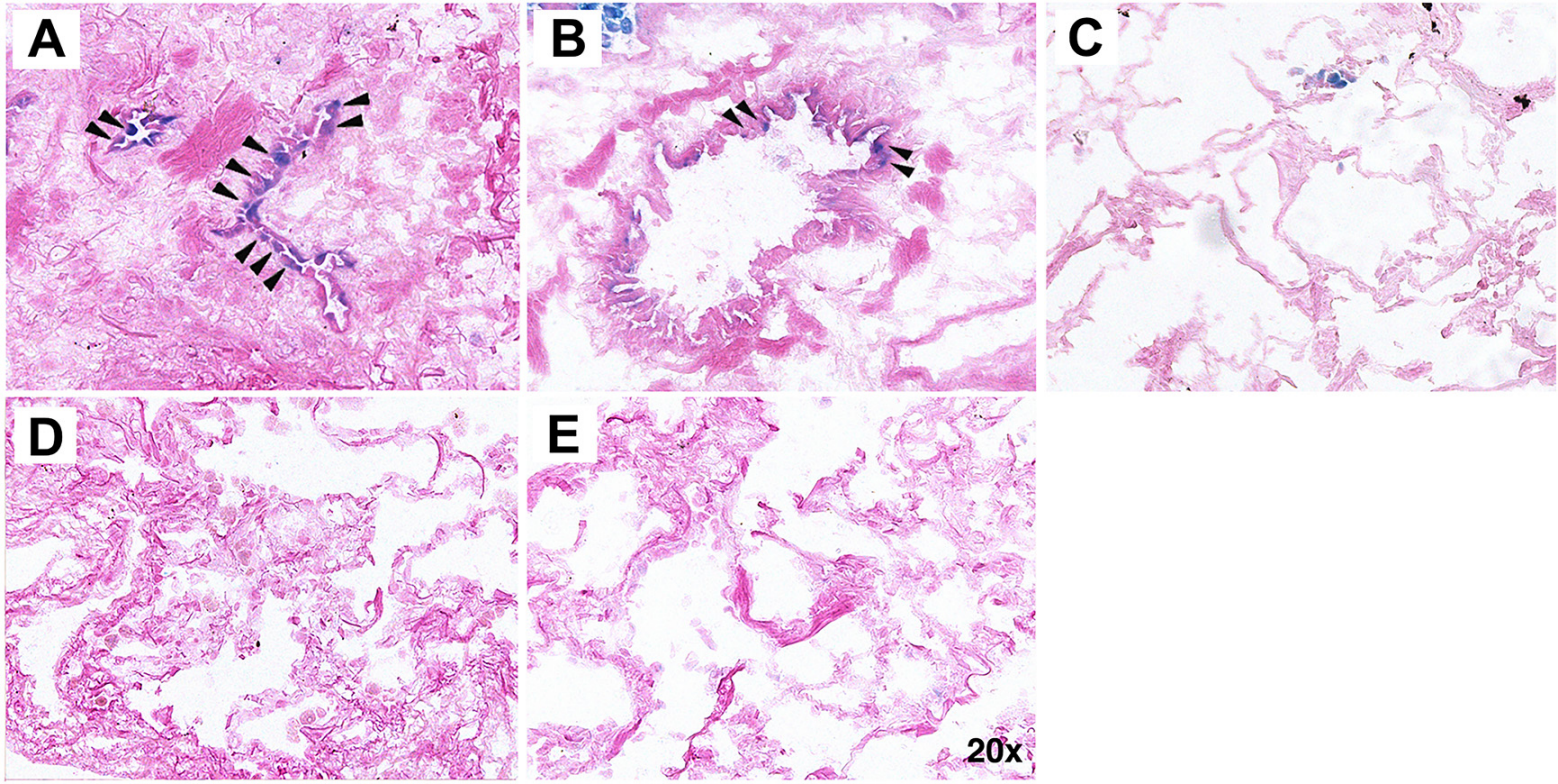
Lung staining for SA-β-galactosidase activity. (A and B) 5 μM sections of lung from of patients with IPF, (C) normal donor controls, (D) scleroderma, or (E) hypersensitivity pneumonitis were stained for SA-βgal activity. Note the blue staining epithelial cells in IPF lung (A and B, arrows) but not normal individuals, or patients with scleroderma or hypersensitivity pneumonitis. Images are representative of staining of tissue from 8 patients with IPF, 14 control subjects, 5 patients with scleroderma, and 8 patients with hypersensitivity pneumonitis.

Recent reports have associated mutations in *TERT* or *TERC* [4, 29, 30] and the presence of short telomeres in type II AECs [4] to the development of IPF. Because telomere shortening can lead to senescence [31], these findings suggest senescence may occur in the type II AECs in IPF lung. To evaluate for senescence, frozen lung sections were stained for SA-βgal, the most common method used to detect senescent cells in tissues. [11] Importantly, a subpopulation of type II AECs in IPF lungs stained for SA-βgal (blue cells, Fig 1A and 1B and S1 Fig). SA-βgal tended to be less prominent in regions of more normal appearing lung in IPF patients (Fig 1B and S2 Fig). SA-βgal was not detectable in type II AECs of normal lungs (Fig 1C), the lungs of patients with scleroderma (Fig 1D), or hypersensitivity pneumonitis (Fig 1E).

### p16, p21, and p53 immunostaining of IPF lung

**Fig 2:**
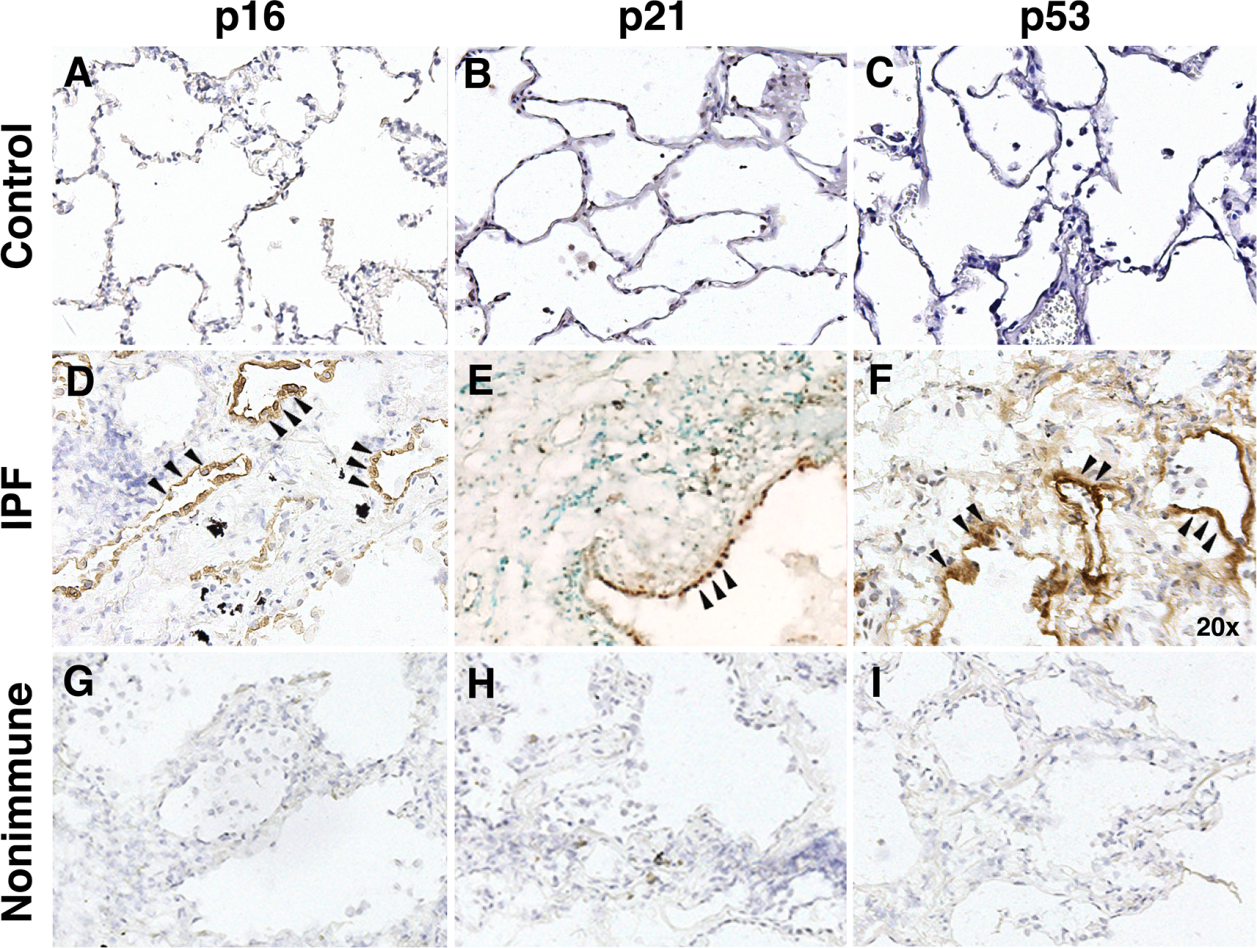
Immunohistochemistry of IPF and normal lung with p16, p21, or p53 antibodies. 5 μM sections of normal lung immunostained for (A) p16, (B) p21, (C) p53 show an absence of staining. In contrast, IPF lung immunostained for (D) p16, (E) p21, or (F) p53 show immunoreactive cells lining airspaces consistent with type II AECs. (G-I) Isotype control for p16, p21, or p53 are shown. Images are representative of staining of tissue from 12 patients with IPF and 10 control subjects.

To confirm IPF type II AECs are senescent, IPF lung sections were immunostained for the senescence markers, p16, p21, and p53. Whereas normal lungs had no immunoreactive cells (Fig 2A, 2B and 2C), type II AECs in tissue sections from IPF patients were immunoreactive for p16, p21, and p53 (Fig 2D, 2E and 2F).

### Expression of senescence markers in isolated type II AECs

**Fig 3:**
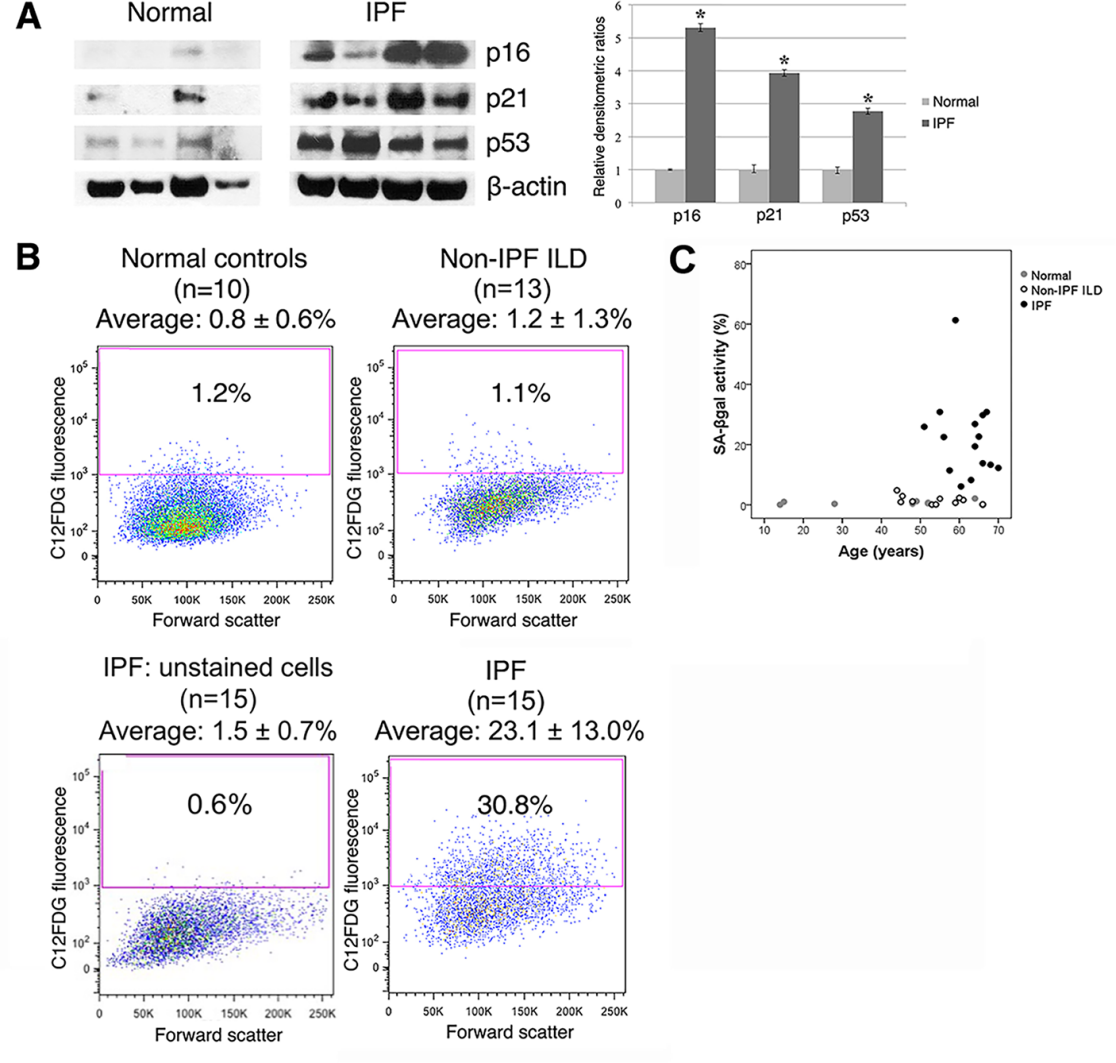
Expression of senescence markers in isolated type II AECs. (A) Immunoblot for p16, p21 and p53 shows higher levels of these proteins in type II AECs isolated from IPF patients relative to those from control subjects. Relative densitometric ratios of p16, p21, and p53 to β-actin are presented by bar graphs (* p < 0.05). (B) SA-βgal activity in type II AECs isolated from IPF lungs was detected in an average of 23.1 ± 13% of cells relative to type II AECs isolated from disease controls (1.2 ± 1.3%), control subjects (0.8 ± 0.6%) and unstatined type II AECs (1.5 ± 0.7%). (C) Scatter plots of age and SA-βgal activity of patients whose type II AECs were analyzed by flow cytometry.

To verify the cells immunoreactive for senescence markers in IPF lung sections are type II AECs, we quantified senescence markers in type II AECs isolated from IPF lung. First, immunoblots were used to show that type II AECs isolated from IPF patients had significantly higher levels of p16, p21, and p53 compared to type II AECs cells isolated from normal controls (Fig 3A). Next, we quantified the population of senescent type II AECs in lung digests using SA-βgal staining to identify senescent cells (S2 and S3 Figs). [24] Importantly, SA-βgal activity was detected in an average of 23.1 ± 13.0% of IPF type II AECs. In contrast, SA-βgal activity was detected in only 0.8 ± 0.6% of type II AECs isolated from normal controls or 1.2 ± 1.3% from non-IPF ILD patients (Fig 3B). When equal numbers of SA-βgal^+^ and SA-βgal^-^ type II AECs were cultured for 10 days, it was found that SA-βgal^+^ type II cells do not expand in culture whereas SA-βgal^-^ populations do expand in culture (S4 Fig), confirming a senescent phenotype. Baseline characteristics of these patients are shown in S2 Table. The mean age of normal controls was lower than the mean age of non-IPF ILD and IPF patients (Fig 3C). However, using regression analysis, age was not a predictor for SA-βgal activity. In contrast, the diagnosis of IPF was a significant predictor for SA-βgal activity (*p* < 0.001).

### miRNA analysis of IPF type II AECs

**Fig 4:**
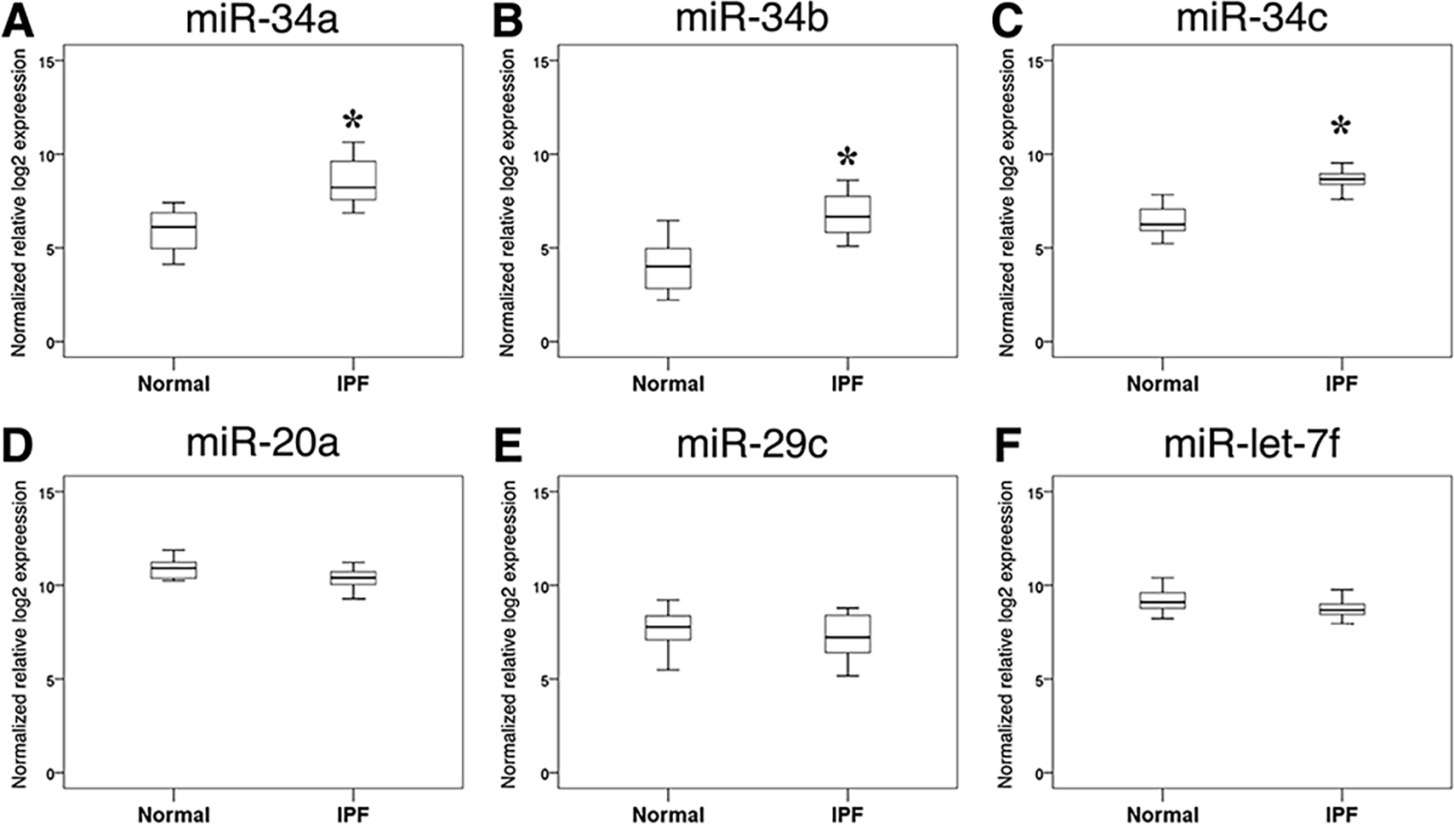
qRT-PCR for miRNAs isolated from type II AECs. Box plots of normalized relative log_2_ expression of miRNAs in type II AECs isolated from IPF patients (n = 15) and control subjects (n = 15). The levels of miR-34a (A), miR-34b (B), and miR-34c (C) were significantly higher in type II AECs isolated from IPF patients compared to those from control subjects (* p< 0.001 by Mann-Whitney U test). The levels of (D) miR-20a, (E) miR-29c, and (F) miR-let-7f were not significantly different in IPF patients relative to control subjects. The boxes are drawn extending from the 75^th^ percentile to the 25^th^ percentile. The horizontal line inside the box represents median values. The whiskers above and below the box delineate the maximum and minimum.

We next sought to identify additional molecular regulators of senescence in IPF type II AECs. Because miRNAs are reported to regulate cellular senescence, [15] a miRNA oligonucleotide array was used to screen for differential expression of 637 miRNAs in type II AECs isolated from 4 IPF lungs and 4 control lungs. There were 22 miRNAs expressed at least 2-fold higher and 7 miRNAs expressed at least 0.5-fold lower in IPF type II AECs (*p* < 0.05 by Student’s t-test, see S3 Table). Of miRNAs reported to regulate senescence, [17, 32–35] three (miR-34a, miR-34b, miR-34c) were elevated in IPF type II AECs and three others (miR-20a, miR-29c, and miR-let-7f) were elevated (1.1–1.9-fold higher, p > 0.1) but not significantly. To validate the candidate SA-miRNAs were differentially expressed, qRT-PCR was used to quantify miR-34a, miR-34b, miR-34c, miR-20a, miR-29c, and miR-let-7f in type II AECs from a larger set of lungs (15 IPF and 15 control). Of these miRNAs, only miR-34a, miR-34b, and miR-34c were confirmed to be elevated in IPF type II AECs (Fig 4A, 4B, 4C, 4D, 4E and 4F). To test whether serum miR34 levels could reflect differential expression of type II AEC in IPF lungs, miR-34a and miR-34b, but not miR-34c, were measured in serum of normal and IPF subjects. miR-34a and miR-34b, but not miR-34c were detectable in serum of control and IPF subjects. However, there was no significant difference in serum miR-34 levels (not shown).

### miRNA overexpression in lung epithelial cells

**Fig 5:**
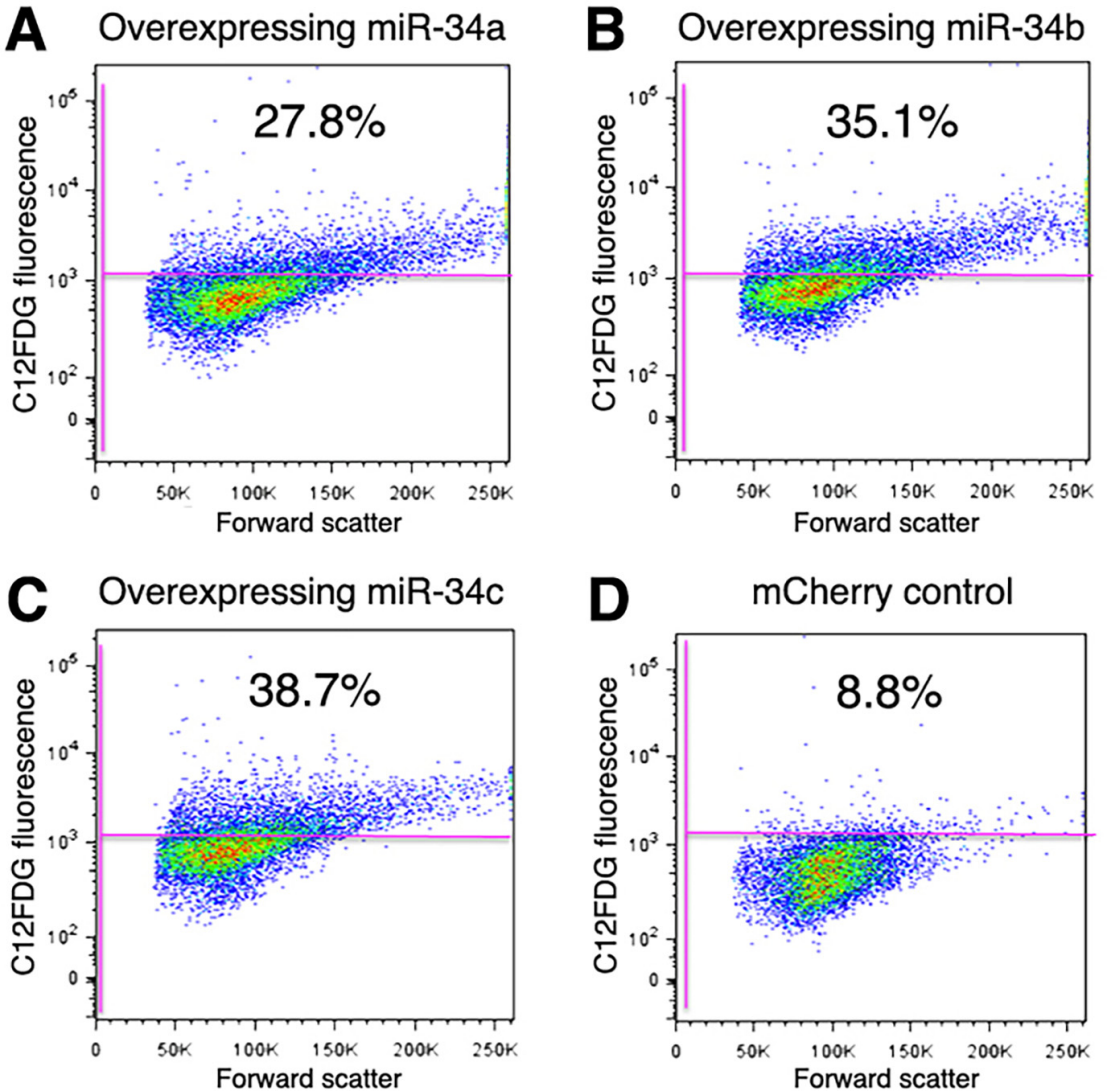
SA-βgal activity detected by flow cytometry in A549 cells overexpressing miR-34a, miR-34b, and miR-34c. The relative percentage of cells with detectable SA-gal activity was measured in A549 cells overexpressing (A) miR-34a, (B) miR-34b, (C) miR-34c, or (D) mCherry control. Note the higher percentage of cells with SA-βgal activity in cells overexpressing miR-34a, miR-34b, or miR-34c compared to treated control cells.

Next we sought to confirm that overexpression of miR-34 family of miRNAs can lead to cellular senescence in lung epithelial cells. To achieve this, lentivirus was used to overexpress miR-34a, miR-34b, or miR-34c in A549 cells. [25] It was found that cells overexpressing miR-34a, miR-34b, or miR-34c had a relative increase in the numbers of cells expressing SA-βgal activity (Fig 5 and S5 Fig). In addition, levels of mRNA expression of p16 and p21 were increased between 1–1.9 fold in A549 cells expressing miR-34s (S4 Table).

### miR-34 targets in type II AECs

**Fig 6:**
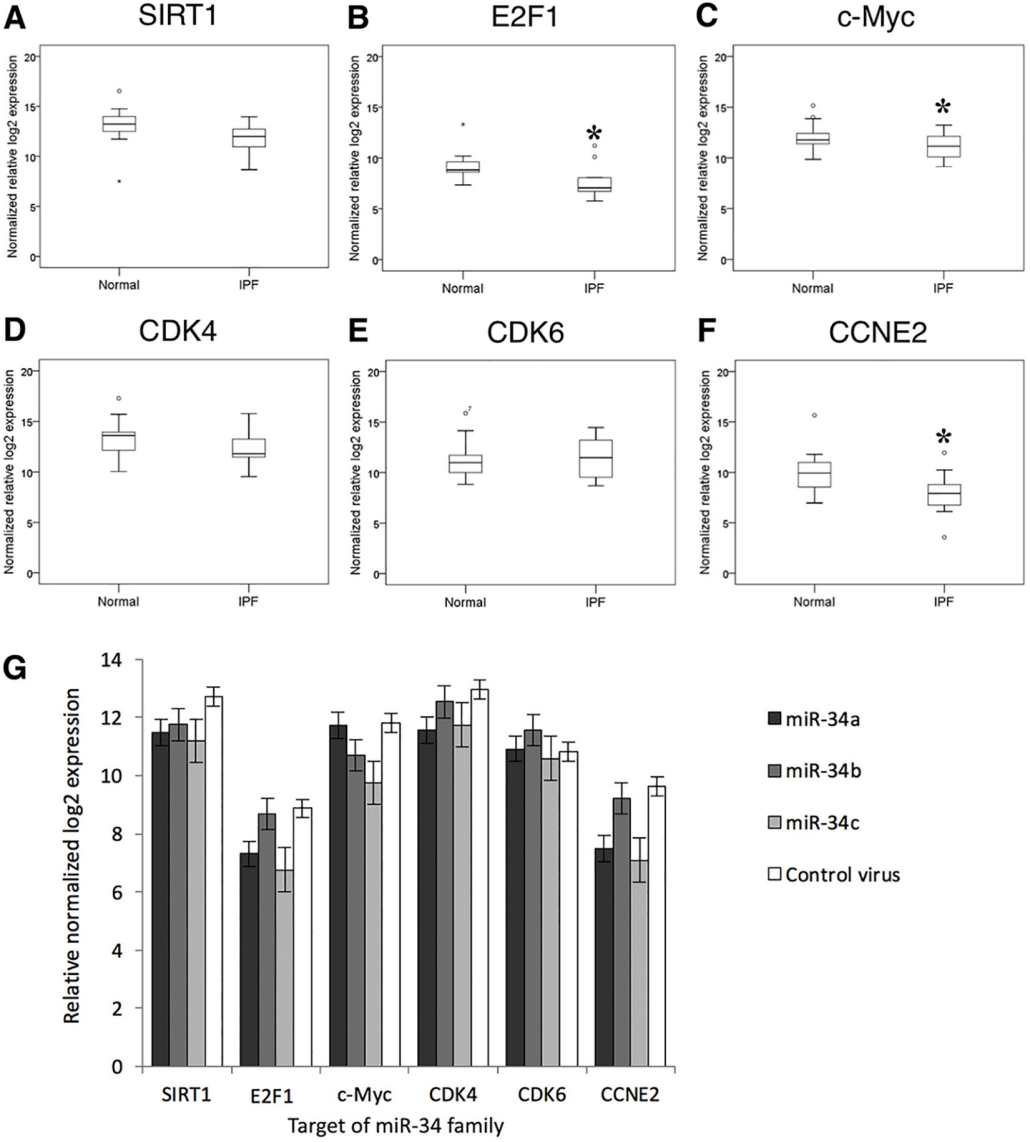
Targets of miR-34 family in isolated type II AECs and A549 cells overexpressing miR-34 family miRNAs. (A-F) Box plots of normalized relative log_2_ expression of SIRT1, E2F1, c-Myc, CDK4, CDK6, and CCNE2 in type II AECs isolated from IPF patients (n = 15) and control subjects (n = 15). The expression levels of (B) E2F1, (C) c-Myc, and (F) CCNE2 were significantly lower in type II AECs isolated from IPF patients compared to those from control subjects (* *p* < 0.05 by Mann-Whitney U test). The boxes are drawn extending from the 75^th^ percentile to the 25^th^ percentile. The horizontal line inside the box represents median values. The whiskers above and below the box delineate the maximum and minimum. (G) Expression levels of candidate targets of miR-34 family were also measured in A549 overexpressing miR-34a, miR-34b, or miR-34c by qRT-PCR.

Several targets of the miR-34 family that mediate a senescent phenotype have been reported including SIRT1, E2F, c-Myc, CDK4, CDK6, and cyclin E2 (CCNE2). [36–39] We used qRT-PCR to examine whether these candidate miR-34 targets were differentially expressed in IPF type II AECs, finding that E2F1, c-Myc, and CCNE2 were significantly lower in IPF type II AECs (Fig 6A, 6B, 6C, 6D, 6E and 6F). Similarly, the expression levels of SIRT1, E2F1, c-Myc, CDK4, and CCNE2 were lower in lung epithelial cells overexpressing miR-34a, miR-34b, or miR-34c (Fig 6G).

## Discussion

Idiopathic pulmonary fibrosis is a progressive lung disease that is associated with chronological aging. Cellular senescence is a pathologic feature of IPF. [7, 40, 41] However, the type of senescent cells and mediators of senescence in IPF have not been fully characterized. The findings reported in this study advance this association by quantification of molecular and phenotypic markers of cellular senescence (p16, p21, p53 and SA-βgal activity) specifically in type II AECs isolated from IPF lungs, and identification of miR-34 miRNAs as mediators of this senescence. These results show that senescence markers were uniquely present in type II AECs of IPF lungs.

Cellular senescence, a condition of stable cell-cycle arrest, is involved in multiple physiologic processes including embryonic development, [42] tumor suppression, [43] tissue repair, [44, 45] and organismal aging. [46] Two senescence pathways have been proposed, the p53-p21-pRb pathway and the p16-pRb pathway. [9, 10] These pathways are activated by various stimuli including oncogenic stress, oxidative stress, DNA damage, or telomere shortening. Stimuli identified in IPF patients that could lead to cellular senescence are telomere shortening or DNA damage. [4, 47] DNA damage signaling leads to activation of ataxia telangiectasia mutated (ATM) kinases that phosphorylate p53. Activation of p53 stimulates transcription of p21 and the Rb protein, leading to cell cycle arrest. [48–50] Analogous to p53, activation of p16 also leads to activation of Rb protein and inhibition of cyclin-dependent kinases. [51] In the present study, we report that p16, p21, and p53 are elevated in IPF lungs. Consistently, SA-βgal activity, the most widely used senescence marker, was present in type II AEC of IPF lungs but not normal lungs or the lungs of scleroderma and hypersensitivity pneumonitis patients. These results show that type II AEC cellular senescence is a unique feature of IPF and is not a general feature of lung fibrosis or normal lung aging.

Digests of human lungs identified type II AECs as a major source of senescent cells in IPF lung. Multiple senescence markers (p16, p21, p53, and SA-βgal activity) were elevated in type II AECs isolated from IPF lungs. Quantifying the proportion of type II AECs with detectable SA-βgal activity showed that on average nearly 23% of the type II AECs are senescent in IPF lungs with proportions as high as 61% in individual patients. Whether the relative percent of senescent type II AECs varies during the course of disease, or correlates with disease progression requires future study.

Recent studies have reported miRNAs that mediate cellular senescence. These miRNAs regulate cellular senescence post-transcriptionally by inhibiting expression of various target genes required for the cell cycle. [16] The majority of these senescence-associated miRNAs (SA-miRNAs) are involved in the p53-p21-pRb or p16-pRb pathways. [9, 10] In this study, miRNA oligonucleotide arrays identified 22 miRNAs whose expression was elevated in type II AECs isolated from IPF lungs. Of these miRNAs, we confirmed the miR-34 family of miRNAs as SA-miRNAs [17, 32, 34] whose expression is up-regulated in type II AECs.

Expression of the miR-34 family of miRNAs is induced by p53 and they have been identified as downstream mediators of p53 regulation of the cell cycle. [17, 39] Activation of p53 induces p21 leading to induction of cell-cycle arrest by inhibiting cyclin E and CDK2 expression. Furthermore, miR-34a also functions as a potent suppressor of cell proliferation by causing downregulation of E2F, which we found to be lower in IPF type II AECs. [34] Activation of miR-34 miRNAs results in multiple phenotypic changes depending on the target it modulates. [17] In this study, targets of miR-34 miRNAs resulting in senescent phenotype were validated by qRT-PCR include downregulation of E2F1, c-Myc, and CCNE2 in IPF type II AECs.

Apart from the role of miR-34 miRNAs in cellular senescence, these miRNAs have also been shown to be required for lung ciliogenesis in mice. Using methods of miR-34 depletion, Song and colleagues reported that miR-34 family members lead to enhanced ciliogenesis. [52] Here we demonstrated the up-regulation of miR-34 family members and elevation of senescence markers in purified type II AECs isolated from IPF patients. There was no evidence of senescence marker expression in multiciliated cells in IPF tissue sections or of contamination of our type II AECs preps with multiciliated cells, limiting the possibility contamination by ciliated cells explains the higher levels of miR34 in IPF AECs. Additionally, we confirmed that A549 cells overexpressing miR-34a, miR-34b, or miR-34c had greater percentage of SA-β-gal activity than control cells.

**Fig 7:**
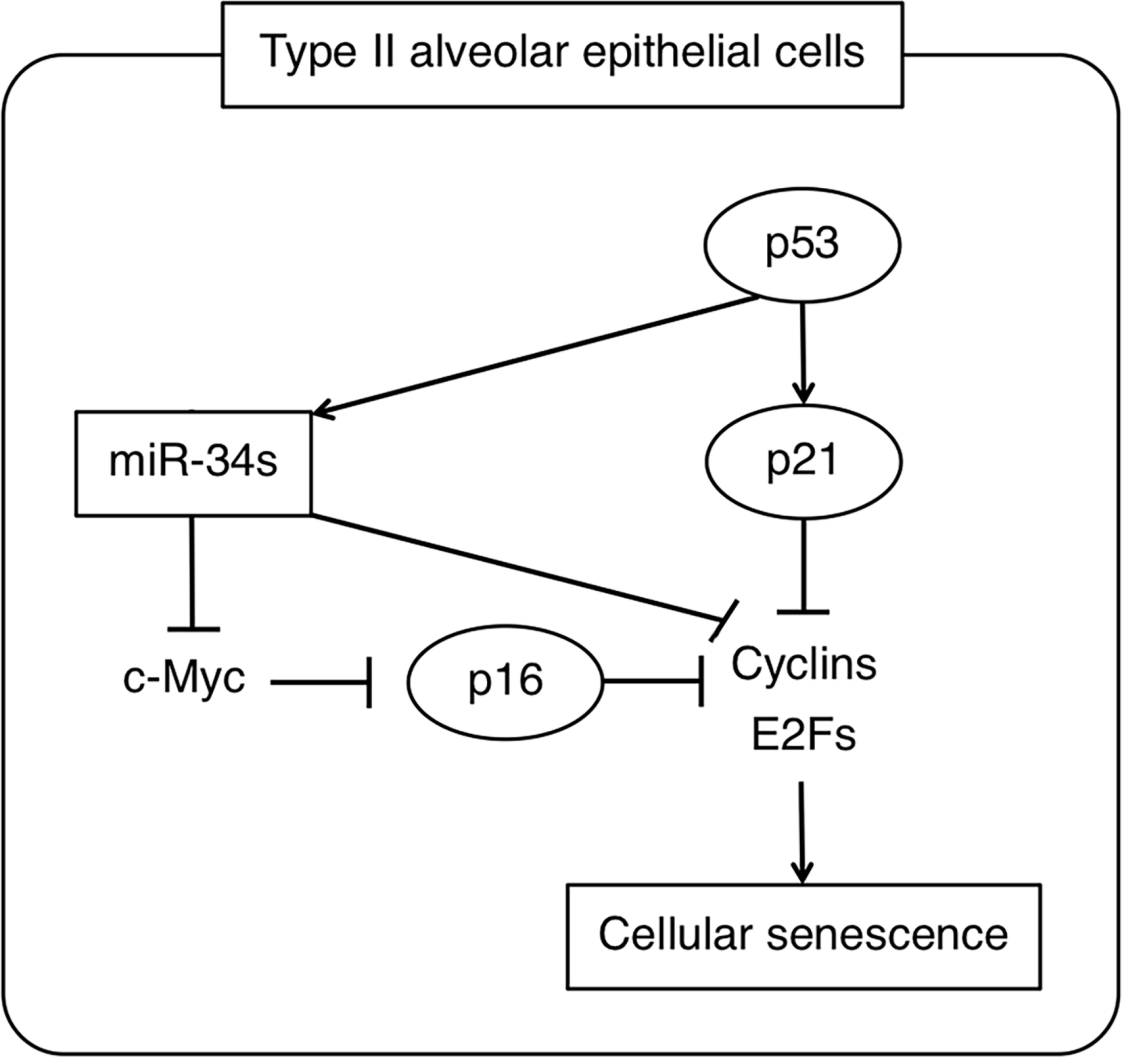
Proposed relationship between miR-34 miRNAs and their targets as mediators of cellular senescence in IPF type II AECs. p53 activation leads to up-regulation of miR-34 family of miRNAs in IPF type II AECs. Elevated miR-34a, miR-34b, or miR-34c levels lead to down-regulation of key target genes, including E2F1, c-Myc, and CCNE2, involved in the cell cycle, leading to cellular senescence.

In summary, these data suggest that up-regulation of miR-34 miRNAs in IPF type II AECs, possibly driven by p53 activation, leads to down-regulation of key targets involved in the cell cycle leading to a senescent phenotype in these cells (Fig 7). Whether their expression in IPF type II AECs contributes to lung fibrosis in addition to senescence, similar to their purported role in age-related cardiac fibrosis, [53] will require additional study.

## Supporting Information

S1 Fig SA-βgal ^+^ and SA-βgal − epithelial cells isolated by flow cytometry, showing that > 90% are immunoreactive to SPC (red flouresence). (TIF)

S2 Fig The lower magnification views of SA-βgal of IPF lungs. (TIF)

S3 Fig Cytospins of SA-βgal ^+^ and SA-βgal − type II epithelial cells isolated by flow cytometry were stained for SA-βgal, confirming that SA-βgal activity is detectable in the SA-βgal ^+^ fraction. (TIF)

S4 Fig Equal numbers of SA-βgal ^+^ and SA-βgal − type II epithelial cells isolated by flow cytometry were cultured in SAGM media for 14 days. Note the increased numbers of SA-βgal−type II epithelial cells compared to SA-βgal ^+^ control cells. Increased numbers was confirmed by counting the cells by flow cytometry (panel C).
(TIF)

S5 Fig A549 cells transfected with lentivirus expressing control vector, or miR-34A, miR-34B, or miR-34C were stained for SA-βgal. Note the positive SA-βgal stain in cells overexpressing miR34s.
(TIF)

S1 Table Primer sequences used for quantitative RT-PCR. (PDF)

S2 Table Baseline characteristics of patients whose type II AECs were analyzed for SA-βgal activity by flow cytometry. (PDF)

S3 Table Profile of differentially expressed miRNAs in IPF type II AECs using miRNA oligonucleotide array. (PDF)

S4 Table Relative p16 or p21 expression in A549 Cells expressing miRNAs. (PDF)
