# Supplementary figures and images for: miR-34 miRNAs Regulate Cellular Senescence in Type II Alveolar Epithelial Cells of Patients with Idiopathic Pulmonary Fibrosis
Source: PLoS One. 2016 Jun 30;11(6):e0158367. doi: 10.1371/journal.pone.0158367 (PMC4928999; doi:10.1371/journal.pone.0158367)

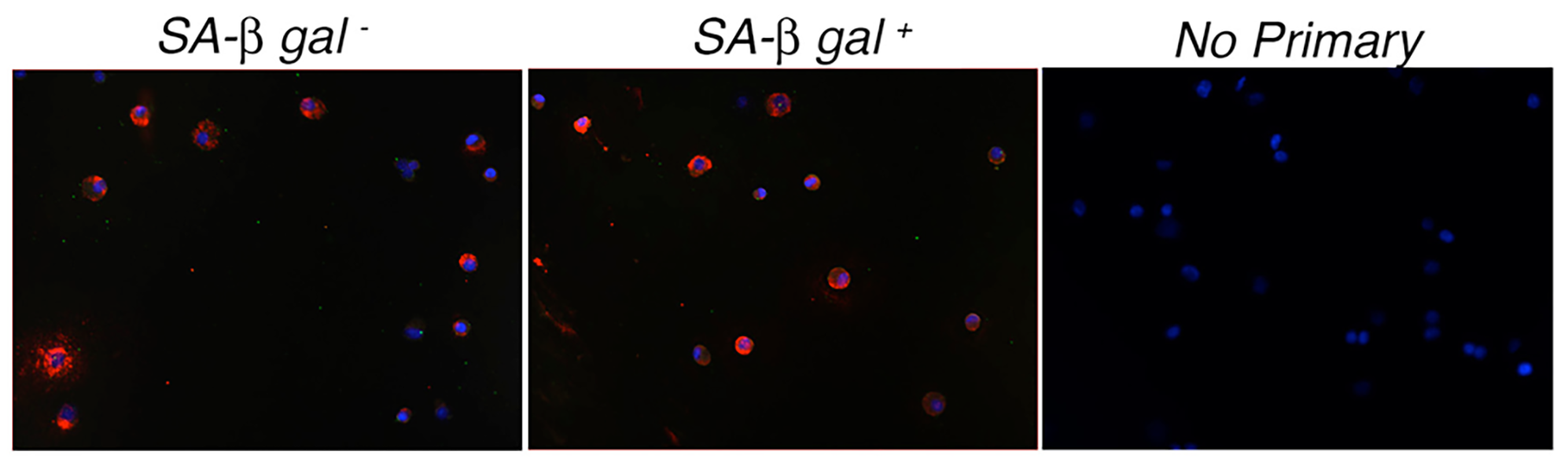

Supplement: S1 Fig — (TIF) [file pone.0158367.s001.tif]

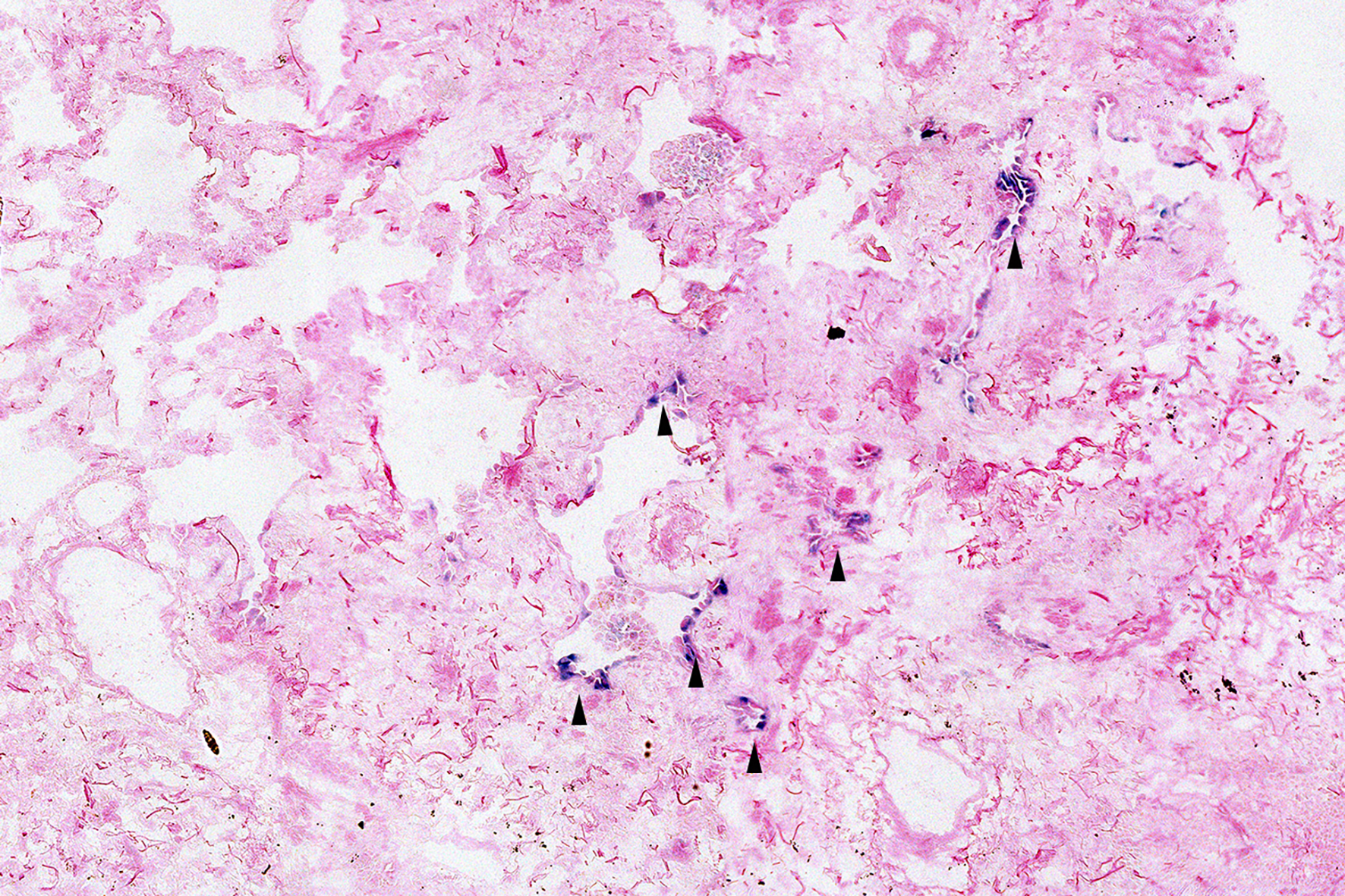

Supplement: S2 Fig — (TIF) [file pone.0158367.s002.tif]

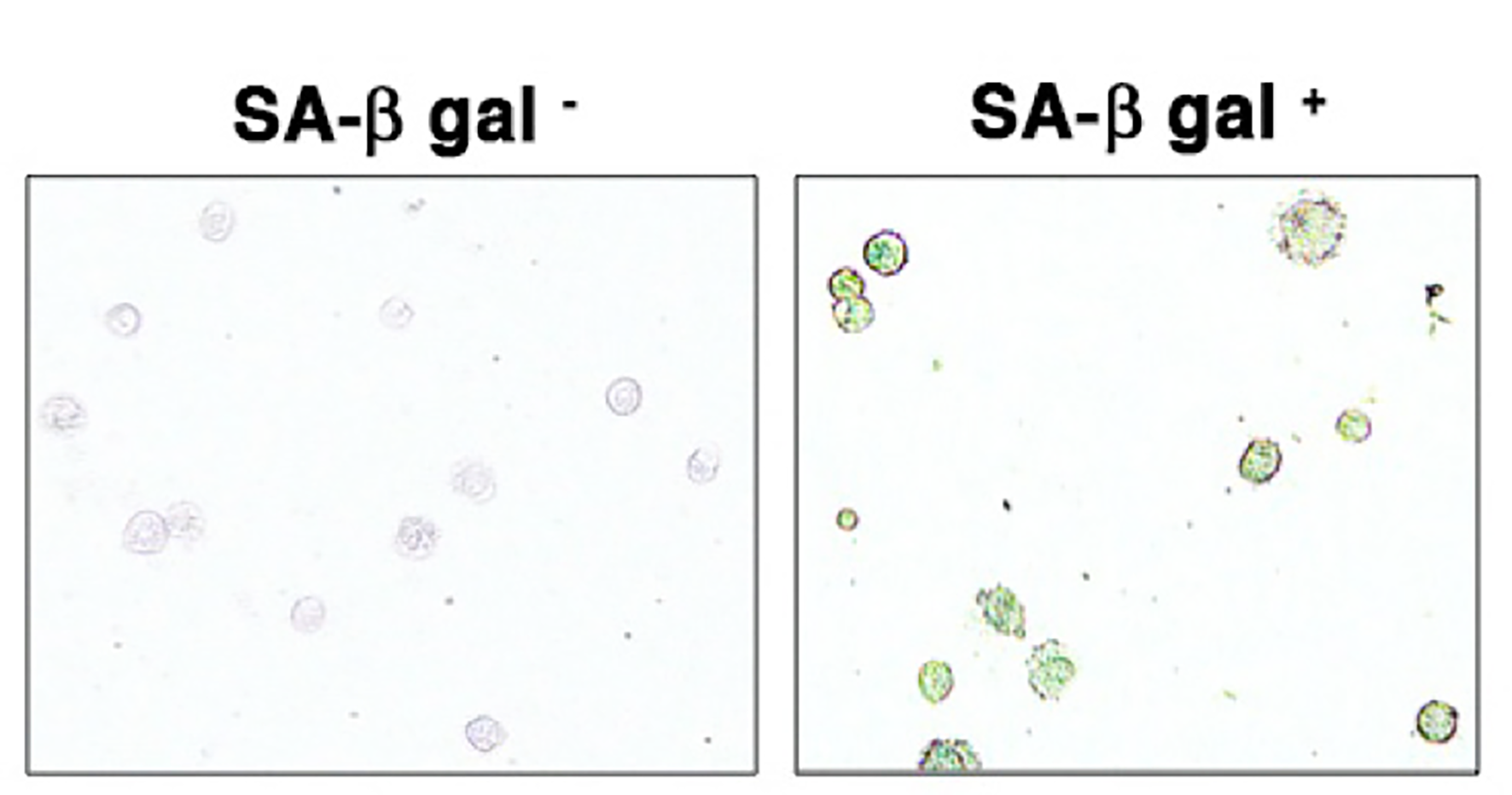

Supplement: S3 Fig — (TIF) [file pone.0158367.s003.tif]

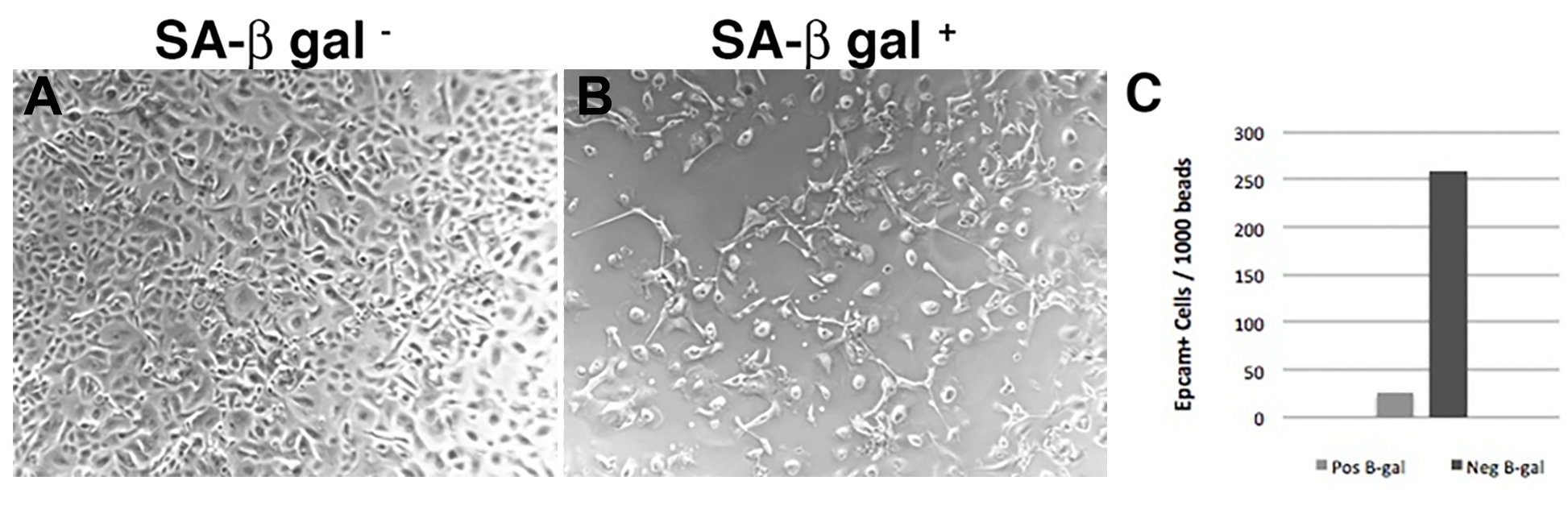

Supplement: S4 Fig — Note the increased numbers of SA-βgal−type II epithelial cells compared to SA-βgal + control cells. Increased numbers was confirmed by counting the cells by flow cytometry (panel C). (TIF) [file pone.0158367.s004.tif]

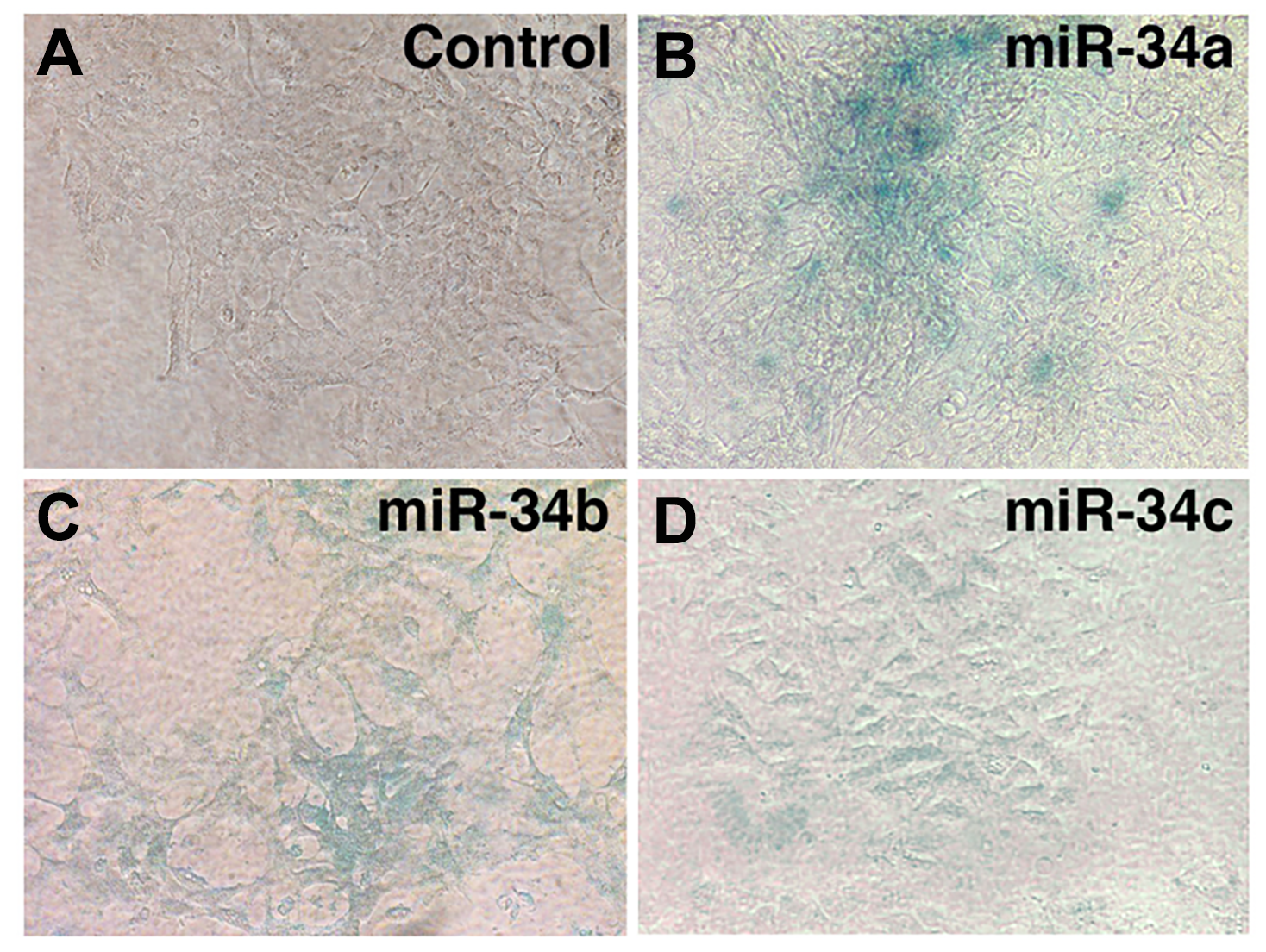

Supplement: S5 Fig — Note the positive SA-βgal stain in cells overexpressing miR34s. (TIF) [file pone.0158367.s005.tif]
