# Supplementary material for: miR-34 miRNAs Regulate Cellular Senescence in Type II Alveolar Epithelial Cells of Patients with Idiopathic Pulmonary Fibrosis
Source: PLoS One. 2016 Jun 30;11(6):e0158367. doi: 10.1371/journal.pone.0158367 (PMC4928999; doi:10.1371/journal.pone.0158367)
Supplement: S1 Table — (PDF) [file pone.0158367.s006.pdf]

S1 Table. Primer sequences used for quantitative RT-PCR.

miRNA or gene name	Primer	Primer sequence
miR-20a	SLR	CTCAACTGGTGTCGTGGAGTCGGCAATTCAGTTGAGCTACCTGC
	Forward	ACACTCCAGCTGGGTAAAGTGCTTATAGTGC
	Probe	TTCAGTTGAGCTACCTGC
miR-29c	SLR	CTCAACTGGTGTCGTGGAGTCGGCAATTCAGTTGAGTAACCGAT
	Forward	ACACTCCAGCTGGGTAGCACCATTTGAAAT
	Probe	TTCAGTTGAGTAACCGAT
miR-34a	SLR	CTCAACTGGTGTCGTGGAGTCGGCAATTCAGTTGAGACAACCAG
	Forward	ACACTCCAGCTGGGTGGCAGTGTCTTAGCT
	Probe	TTCAGTTGAGACAACCAG
miR-34b	SLR	CTCAACTGGTGTCGTGGAGTCGGCAATTCAGTTGAGATGGCAGT
	Forward	ACACTCCAGCTGGGCAATCACTAACTCCAC
	Probe	TTCAGTTGAGATGGCAGT
miR-34c	SLR	CTCAACTGGTGTCGTGGAGTCGGCAATTCAGTTGAGGCAATCAG
	Forward	ACACTCCAGCTGGGAGGCAGTGTAGTTAGCT
	Probe	TTCAGTTGAGGCAATCAG
miR-let-7f	SLR	CTCAACTGGTGTCGTGGAGTCGGCAATTCAGTTGAGAACTATAC
	Forward	ACACTCCAGCTGGGTGAGGTAGTAGATTGT
	Probe	TTCAGTTGAGAACTATAC
miR-103	SLR	CTCAACTGGTGTCGTGGAGTCGGCAATTCAGTTGAGCAGCTGCT
	Forward	ACACTCCAGCTGGGAGCAGCATTGTACAGGG
	Probe	TTCAGTTGAGTCATAGCC

miR-191	SLR	CTCAACTGGTGTCGTGGAGTCGGCAATTCAGTTGAGCAGCTGCT
	Forward	ACACTCCAGCTGGGCAACGGAATCCCAAAAG
	Probe	TTCAGTTGAGCAGCTGCT
SIRT1	Reverse	TGAGGCACTTCATGGGGTATGG
	Forward	TCCTAGGTTGCCCAGCTGATGAA
c-Myc	Reverse	CTTCTCTCC GTCCTCGGATTCT
	Forward	GAAGGTGATCC AGACTCTGACCTT
CDK4	Reverse	CTGGTGTTTGAGCATGTAGACC
	Forward	AAACTGGCGCATCAGATCCTT
CDK6	Reverse	TGCACAGTGTCACGAACAGA
	Forward	ACCTCGGAGAAGCTGAAACA
E2F1	Reverse	ATGTTTTCCTGTGCCCTGAG
	Forward	ATCTGTGGTGAGGGATGAGG
CCNE2	Reverse	CTATTTGGCTATGCTGGAGG
	Forward	TCTTCGGTGGTGTCATAATG
β-actin	Reverse	CTCGTTAATGTCACGCAC
	Forward	CGTCTTCCCCTCCATCG
GAPDH	Reverse	CGCCCCACTTGATTTTGGA
	Forward	TTGCCATCAATGACCCCTTCA

SLR = stem-loop reverse
