# Supplementary material for: miR-34 miRNAs Regulate Cellular Senescence in Type II Alveolar Epithelial Cells of Patients with Idiopathic Pulmonary Fibrosis
Source: PLoS One. 2016 Jun 30;11(6):e0158367. doi: 10.1371/journal.pone.0158367 (PMC4928999; doi:10.1371/journal.pone.0158367)
Supplement: S2 Table — (PDF) [file pone.0158367.s007.pdf]

S2 Table. Baseline characteristics of patients whose type II AECs were analyzed for SA-βgal activity by flow cytometry.

Baseline characteristics	Normal (n, %)	Non-IPF ILD (n, %)	IPF (n, %)
Number of patients	10	13	15
Age, year*	40 ±19 (14-64)	55 ± 8 (44-66)†	62 ± 6 (51-70)‡
Sex, male	5 (50)	6 (46.2)	13 (86.7)
Smoking	ND	8 (61.5)	10 (66.7)
FVC, % predicted*	ND	52.1 ± 15.1 (27-74)	52.5 ± 12.9 (33-77)
DLCO, % predicted*	ND	17.0 ± 7.3 (8-30)	24.7 ± 8.4 (12-39)

* Mean ± SD (range); † $p = 0.020$, compared to control group; ‡ $p < 0.001$, compared to control group; IPF = idiopathic pulmonary fibrosis; ILD = interstitial lung disease; FVC = force vital capacity; DLCO = diffuse capacity for carbon monoxide; ND = not done
