# Supplementary material for: miR-34 miRNAs Regulate Cellular Senescence in Type II Alveolar Epithelial Cells of Patients with Idiopathic Pulmonary Fibrosis
Source: PLoS One. 2016 Jun 30;11(6):e0158367. doi: 10.1371/journal.pone.0158367 (PMC4928999; doi:10.1371/journal.pone.0158367)
Supplement: S3 Table — (PDF) [file pone.0158367.s008.pdf]

S3 Table. Profile of differentially expressed miRNAs in IPF type II AECs using miRNA oligonucleotide array.

Higher in IPF type II AECs (> 2-fold, unadjusted $p < 0.05$)		Lower in IPF type II AECs (< 0.5-fold, unadjusted $p < 0.05$)	
miRNA name	Fold difference	miRNA name	Fold difference
miR-15a	2.085	miR-197	0.426
miR-21	4.320	miR-210	0.406
miR-23a	2.050	miR-886	0.399
miR-27a	2.132	miR-939	0.472
miR-27b	2.333	miR-1183	0.451
miR-31	13.453	miR-1229	0.481
miR-34a	7.823	miR-1268	0.446
miR-34b	7.514		
miR-34c	16.475		
miR-100	2.578		
miR-138	3.772		
miR-149	3.766		
miR-193b	2.025		
miR-200a	2.224		
miR-200b	2.439		
miR-205	14.514		
miR-345	2.024		

Higher in IPF type II AECs (> 2-fold, unadjusted $p < 0.05$)		Lower in IPF type II AECs (< 0.5-fold, unadjusted $p < 0.05$)	
miRNA name	Fold difference	miRNA name	Fold difference
miR-429	2.870		
miR-449a	6.006		
miR-449b	5.413		
miR-449c	3.900		
miR-551b	4.480		

IPF = idiopathic pulmonary fibrosis; AECs = alveolar epithelial cells
