# Supplementary material for: miR-34 miRNAs Regulate Cellular Senescence in Type II Alveolar Epithelial Cells of Patients with Idiopathic Pulmonary Fibrosis
Source: PLoS One. 2016 Jun 30;11(6):e0158367. doi: 10.1371/journal.pone.0158367 (PMC4928999; doi:10.1371/journal.pone.0158367)
Supplement: S4 Table — (PDF) [file pone.0158367.s009.pdf]

S4 Table. Relative p16 or p21 expression in A549 Cells expressing miRNAs*

miRNA	Fold Increase p16	Fold Increase p21
miR34a	1.0	1.1
miR34b	1.3	1.4
miR34c	1.9	1.8

*Relative p16 and p21 levels quantified by qRT-PCR in A549 cells expressing either miR34b or miR34c.
